# SGHRP: Secure Greedy Highway Routing Protocol with authentication and increased privacy in vehicular ad hoc networks

**DOI:** 10.1371/journal.pone.0282031

**Published:** 2023-04-06

**Authors:** Edris Khezri, Esmaeil Zeinali, Hadi Sargolzaey

**Affiliations:** Faculty of Computer and Information Technology Engineering, Qazvin Branch, Islamic Azad University, Qazvin, Iran; University College of Engineering Tindivanam, INDIA

## Abstract

VANETs are networks of connected intelligent vehicles that can communicate with each other, as well as with infrastructure and fixed roadside equipment. As a result of the lack of fixed infrastructure and open-access environment, security is crucial when sending packets. Secure routing protocols have been proposed for VANETs, but most are focused on authenticating nodes and creating a secure route, without considering confidentiality after the route is created. Using a chain of source keys validated by a one-way function, we have proposed a secure routing protocol called Secure Greedy Highway Routing Protocol (GHRP), which provides increased confidentiality over other protocols. As part of the proposed protocol, the source, destination, and intermediate nodes are authenticated using a hashing chain in the first stage, and in the second stage, one-way hashing has been used to increase data security. In order to resist routing attacks such as black hole attacks, the proposed protocol is based on the GHRP routing protocol. The proposed protocol is simulated using the NS2 simulator, and its performance is compared with that of the SAODV protocol. Based on the simulation results, the proposed protocol performs better than the mentioned protocol in terms of packet delivery rate, overhead, and average end-to-end delay.

## 1. Introduction

**Fig 1:**
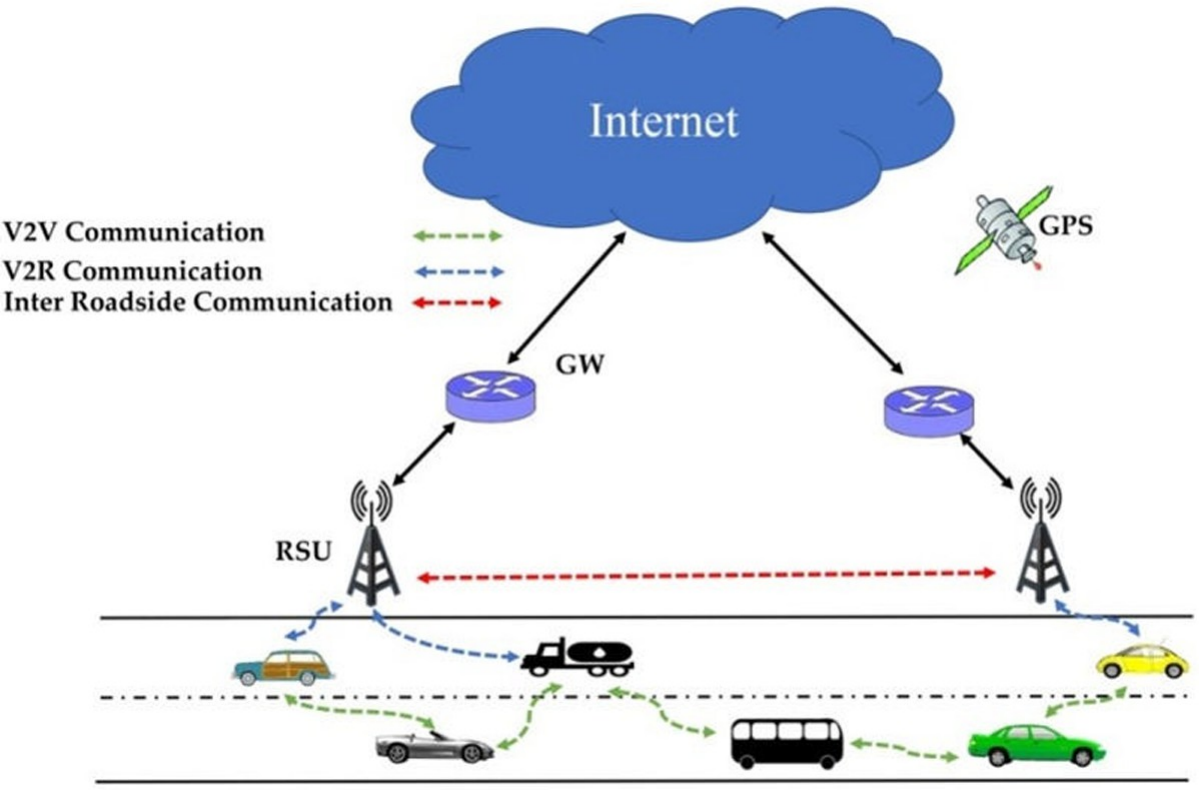
Vehicular ad hoc networks [1].

Vehicular ad hoc network (VANET) is a wireless network in which the vehicles equipped with wireless interface can communicate with each other or fixed roadside equipment (Fig 1) [1–3].

Vehicular Ad hoc Networks research is not comprehensive, there are still challenges that remain unresolved, and more research is needed in this area. In communication environments, most routing protocols focus on the urban environment and less on the highway environment, for example. A number of other challenges should also be considered, including highly dynamic topologies and high mobility, continuous network interruptions, movement route prediction and modeling, diverse communication environments, fault tolerance, distribution networks, security and confidentiality [1, 4–15]. Route discovery and maintenance are particularly important in Vehicular Ad hoc Networks because of their unstable nature. Data packets are routed from their source to their destination in order to ensure reliability. Another goal of routing is to minimize delays [3, 16].

Security is an essential component for performing simple network tasks such as routing and packet forwarding. As all nodes in vehicular ad hoc networks perform this task, security problems are inherent because nodes cannot be trusted to perform their main and vital tasks properly. When there is an initial trust relationship between the nodes of a network, an authentication entity can ensure the proper functioning of critical network functions. There are, however, some special scenarios, such as military and cooperative networks, in which a common trusted center manages the network and impenetrable hardware is required. Authentication in large networks, however, requires key management. Managed environments are those where a common trusted center manages keys [17–25]. A network node that does not have impenetrable hardware and authentication infrastructure, such as in gaming environments without a trusted center to control the network, can compromise the credibility of the network. Besides the correct implementation of the network tasks, each node must also take on a share of these tasks and perform them correctly [17]. Various routing protocols have been proposed for secure routing in vehicular ad hoc networks, including SAODV, Ariadne, ARAN, SEAD, and ECDSA [26]. Most of these existing methods aim to establish security in routing, authentication of nodes from source to destination, but each protocol only focuses on a specific security service. Additionally, each of these protocols unintentionally increases routing overhead in the network and is vulnerable to routing attacks such as black holes. In terms of packet delivery rate, average end-to-end delay, and routing overhead, the proposed protocol outperforms the compared SAODV protocol. The next parts of the paper are organized in the following way: black hole attack in section 2, related work in section 3, GHRP protocol in section 4, proposed protocol in section 5, comparison of the proposed protocol simulation with other SAODV and GHRP protocols in section 6, and finally conclusions, challenges, and future work are discussed in section 7.

## 2. Black hole attack

VANETs are extremely vulnerable to security attacks due to their high dynamism, open access medium, distributed infrastructure, and protocol design problems. Security attacks such as denial-of-service (DoS), Sybil attacks, wormhole attacks (WHA), impersonation attacks, and black hole attacks (BHA) have made them vulnerable. These attacks can compromise VANET applications and services. In a BHA, all network traffic is directed to a specific node, where it disappears like matter in a black hole. It is therefore called a black hole because of the nature of this particular node [7, 12, 27–34]. When a malicious node receives an RREQ packet from a source node, it quickly responds with a fake RREP without checking its routing table. Upon receiving the fake RREP packet, the source node deceptively considers it as an optimal route and forwards data packets towards the black hole. A BHA drops data packets instead of forwarding them, resulting in a decrease in overall network security and performance, as well as disrupting the sharing of network information. Emergency notifications and warning changes may be contained in these packets, which must be delivered quickly and within a specific timeframe. In highly dynamic vehicular ad hoc networks, dropping such packets can result in road accidents, traffic jams, and road casualties [35, 36]. Since BHA is one of the most serious attacks in VANETs, our aim in this study is to present a new and efficient secure routing protocol by authenticating nodes using a hash chain in the route creation phase and using one-way hash functions to increase data security after route creation to counter the BHA attack.

## 3. Related work

In VANETs, routing protocols lacking encryption mechanisms make them vulnerable to routing attacks; however, safety protocols have been designed based on these protocols. The purpose of this section is to provide a brief introduction to some of these protocols.

### 3.1. SAODV routing protocol

SAODV is an extension of the AODV protocol for routing discovery and for providing security features such as integrity, authentication, and non-denial of service [35, 37–39]. The SAODV protocol uses public key cryptography and AODV for routing. All intermediate nodes validate the routing packet in this protocol. As part of the SAODV protocol, two digital signature features are used to ensure that packets are not tampered with, and a hash chain is used to authenticate the route hop count.

### 3.2. ARAN routing protocol

ARAN’s routing protocol [40] is based on AODV. On receiving a certification request from a CA, a third party called a certificate authority (CA) sends a signed certificate to the nodes. Secure route detection is authenticated using asymmetric encryption techniques, and the route is cleared using timestamp [26, 41].

### 3.3. SEAD routing protocol

The SEAD routing protocol [42] is based on the DSDV protocol. To validate random numbers and hop count in the routing table, this protocol uses one-way hashing functions. The protocol also uses symmetric encryption with a shared key between the source and destination [26].

### 3.4. Ariadne routing protocol

YihChun et al. proposed the Ariadne routing protocol in 2005. This protocol validates packets and nodes using three methods: shared keys between two pairs of nodes, TESLA-based shared keys between end nodes, and digital signatures [26, 41].

### 3.5. ECDSA routing protocol

The ECDSA [43] protocol uses a digital signature. Additionally, ECDSA uses hashes and related symmetric key operations to ensure the authenticity and protection of digital signatures. As soon as both sender and receiver agree on the parameters for the elliptical curve domain, it can be initiated [26, 41].

## 4. GHRP routing protocol

**Fig 2:**
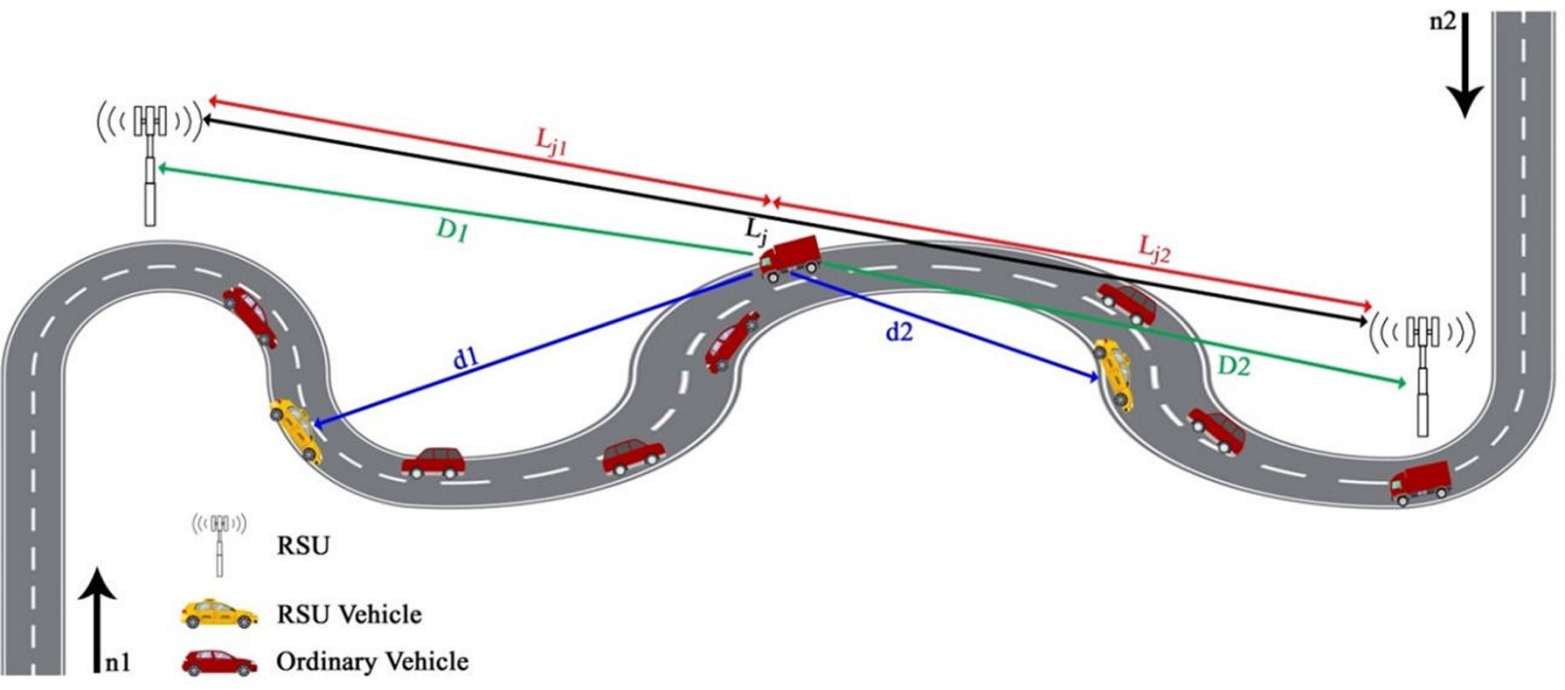
GHRP protocol [3].

The GHRP protocol was introduced by Edris et al. in 2022 [3]. Data transfer protocol Greedy Highway Routing Protocol is designed to distribute information on highways with two-way traffic in the opposite direction (Fig 2). This protocol uses fixed and mobile RSUs to disseminate information. By identifying accident-prone points and installing fixed RSUs in those places, the number of fixed RSUs is minimized to minimize the cost problem, and by using mobile RSUs, which are intercity public transportation vehicles, the entire route can be covered so the total information can be obtained. Reduce the end-to-end delay by aggregating the path (increasing packet delivery rate). An IEEE 802.11p interface and a 4G interface are provided by the On-Board Unit (OBU) of every car that is used as a mobile RSU. For communication with conventional vehicles, IEEE 802.11p is used, and for communication with RSUs (fixed and mobile), 4G is used. As a rule of thumb, 20% of the total number of nodes are considered RSUs. GPS-enabled vehicles are assumed to be aware of their location using the GHRP protocol, and a digital map with road traffic conditions is installed in each vehicle.

The GHRP routing protocol selects the shortest route between source and destination to reduce delay. By estimating the number of steps between the source and the destination, the shortest possible route between the source and the destination is selected. The steps necessary to reach the nearest fixed or moving RSU are calculated as follows:

- If *d*′ ≤ *R* then we have (Eqs 1 and 2):
$${\text{C}}_{\text{j}}=\frac{{\text{L}}_{\text{j}}}{{\text{N}}_{j_{2}k}^{'}\times \text{R}}$$ (1)
$$\text{T}={\text{C}}_{\text{j}}\times {\text{t}}_{\text{d}}$$ (2)
  *d*′ is the average distance between ordinary vehicles from the source to the first RSU, *R* is the coverage radius of each vehicle, *C*_*j*_ is the number of steps to reach the nearest fixed or moving RSU, *T* is the time it takes to reach the nearest fixed or moving RSU, *L*_*j*_ is the length of the jth road section, $N_{j_{2}k}^{'}$ is the number of RSUs in the *L*_*j*_ section, and *t*_*d*_ is the time it takes for two vehicles to send packets.
- If *d*’>R, then we have (Eq 3 and 4):
$${\text{C}}_{\text{j}}={\text{M}}_{j_{2}k}^{'}-1$$ (3)
$$\text{T}=(\sum_{\text{i}=1}^{{\text{M}}_{j_{2}k}^{'}-1}\frac{{\text{V}}_{\text{i}}}{{\text{d}}_{\text{ij}}})+({\text{C}}_{\text{j}}\times {\text{t}}_{\text{d}})$$ (4)
  $M_{j_{2}k}^{'}$ is the number of ordinary vehicles between the source and the first RSU, *V*_*i*_ is the speed of vehicle i, and *d*_*ij*_ is the distance between two vehicles i and j. In the case that *D*_1_ = *D*_2_ (the distance from the source vehicle to the fixed RSU behind it is called *D*_*1*_ and the distance from the source vehicle to the fixed RSU in front is called *D*_*2*_), because the number of steps will be equal, a route with the longest lifetime will be chosen among the available routes. In order to calculate the lifetime, we follow these steps:
  - Both vehicles should be in the same direction, and the speed of the front vehicle should be more. The following equation (Eq 5) calculates the route’s lifetime:
$$LifeTime_{link}=\frac{R-|d_{ij}|}{\left|V_{i}-V_{j}\right|}$$ (5)
  - Both vehicles should be in the same direction, and the speed of the front vehicle must be less. Eq 6 calculates the lifetime of the route in this case:
$$LifeTime_{link}=\frac{R+|d_{ij}|}{\left|V_{i}-V_{j}\right|}$$ (6)
  - The vehicles move in the opposite direction. According to Eq 7, the route’s lifetime is calculated as follows:
$$LifeTime_{link}=\frac{R+|d_{ij}|}{V_{i}+V_{j}}$$ (7)

In this equation, *R* is the radius of each vehicle, *d*_*ij*_ is the distance between vehicle i and vehicle j, *V*_*i*_ is the speed of vehicle i, and *Vj* is the speed of vehicle j.

## 5. Proposed protocol

There are some security issues with the GHRP protocol that result in it being vulnerable to attacks like denial of service (DoS), Sybil attacks, wormhole attacks (WHAs), impersonation attacks and black hole attacks (BHAs). We propose a new secure routing protocol named SGHRP (Secure Greedy Highway Routing Protocol) to authenticate nodes using hash chains and use one-way hash functions to increase data security after routes are created.

One-way hashes are mathematical functions that convert variable-length input strings into fixed-length binary sequences. Moreover, a one-way hash function is designed in such a way that it is difficult to reverse the process, namely to find a string that hashes to a given value. As shown in Table 1, the proposed protocol requires the following parameters.

**Table 1:** Protocol parameters used in SGHRP.

**RDP**	Route request package
**RRP**	Route Response Packet
**REP**	Route Error Packet
**Max_Hash**	Maximum Hashing
**H_S_**	Sender’s random number
**T**	Validity time of the certificate
**TTL**	Maximum hop Count
**Cert_N_**	N node certificate
**IP_D_**	IP address of the destination node
**IP_S_**	IP address of the source node
**Pv__N_**	The private key of node N
**Pub__N_**	The public key of node N
**T_NOW_**	The current time of source
**k_i_**	The key of the time period is i
**T_i_**	The start time of the time period i
**T_int_**	The time interval of each period
**F(k_i_)**	One-way hash function for k_i_ validation
**P_i_**	Packets received in the i-th time interval
**K_SD_**	Shared key between two source and destination nodes

A hash chain is used in the SGHRP protocol to validate the hop count of routing packets (route request packets and route response packets) by intermediate, source, and destination nodes. This method prevents interference attacks by middle nodes.

A hash chain is formed by applying the one-way hash function to the random number generated by the source. As a first step, the source node generates the random number N_s_ and sets it as the initial value of the hashing chain (Eq 8).


$$Hash=h(N_{s})$$ (8)

Afterward, it sets the maximum hop count equal to the packet’s lifetime (Eq 9):

Max_Hop_Count=TTL (9)

By using the TTL of the time of applying the hashing function, it calculates the maximum amount of hashing (Eq 10):

$$Max\_Hah=h^{TTL}(N_{s})$$ (10)

Routing packets are authenticated by intermediate nodes based on the maximum hop count in the packet and the maximum hash value (Eq 11):

$$Max\_Hash=h^{TTL-Hop\_Count}(Hash)$$ (11)

The routing packet contains a packet containing the time information related to increasing privacy. Each intermediate node receives the routing packet and calculates the new hash chain value and MAC value of the packet and replaces them with their previous value. By using their shared keys with the source node, intermediate nodes calculate the MAC. As soon as these intermediate nodes receive the route response, they insert the key into the packet so that the source node can authenticate them.

**Fig 3:**
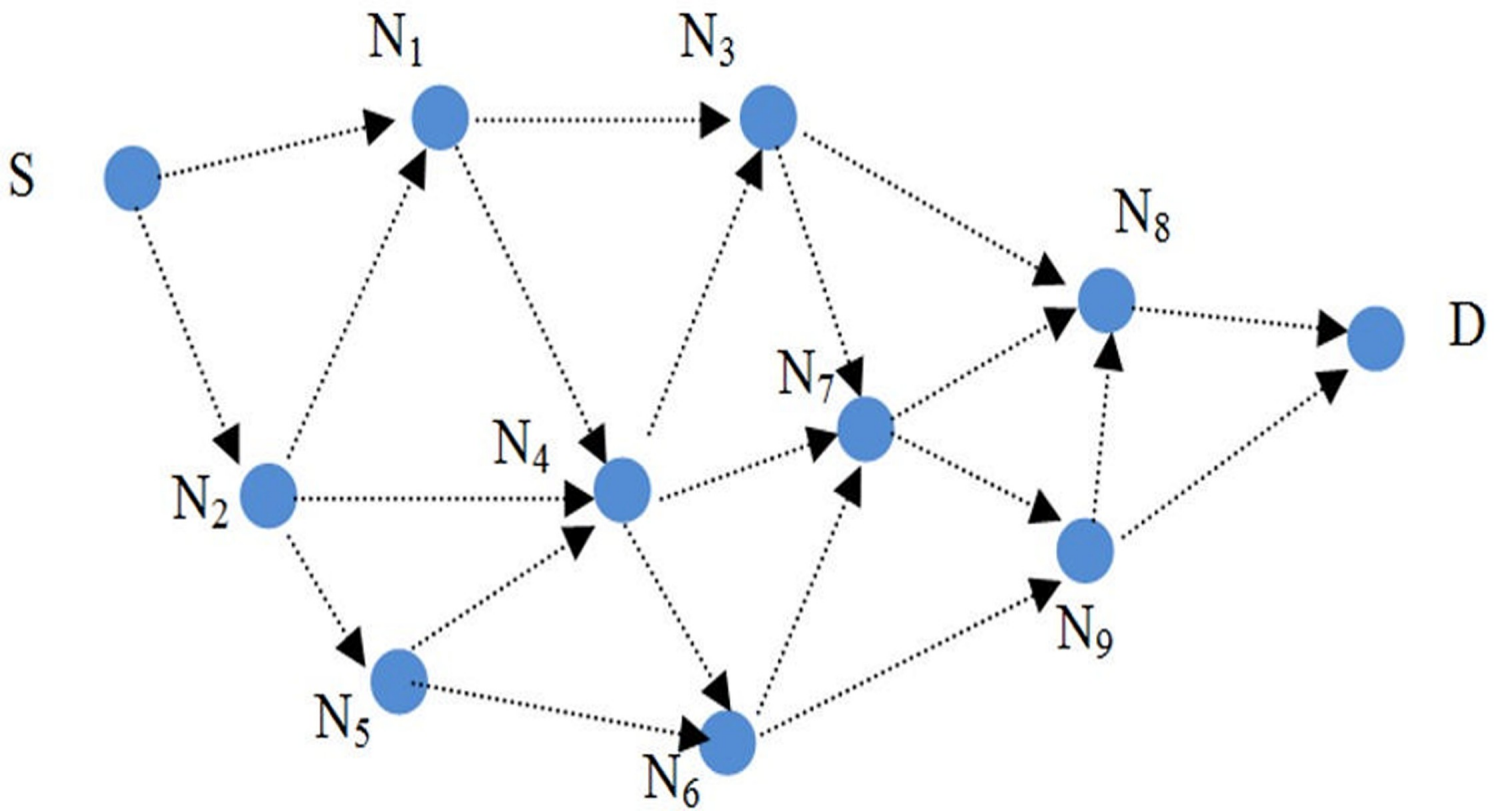
The proposed protocol’s routing.

A VANET network with n nodes is shown in Fig 3. Communication between nodes within radio range of each other can be accomplished in a one-hop, but communication between nodes outside of radio range is accomplished through intermediate nodes. It is assumed that node S intends to create a route to node D. Below are described the steps of the protocol, including route request, route response, route error, and privacy enhancement.

### 5.1. Rout discovery packet phase in SGHRP protocol

The protocol assumes that the nodes have a shared key. According to Fig 3, the source node intends to create a route between itself and the destination. This is accomplished by creating a routing packet with the following format (Eq 12).


$$\left[\text{RDP},{\text{N}}_{\text{s}},{\text{IP}}_{\text{D}},\text{TTL},\text{Max\_hash},\;{\left[\text{P}\right]}_{{\text{K}}_{\text{SD}}},\text{Hop\_count,}\;{\text{h}}_{0}\right]$$ (12)

The initial value of the hashing chain in this package is h_0_, which can be determined by Eq 13:

$${\text{h}}_{\text{0}}=\text{Hash}(\{{\text{RDP,N}}_{\text{s}}\text{,}\;{\text{IP}}_{\text{D}}\text{,}\;\text{TTL}\})$$ (13)

P packets are placed in routing packets to change encryption keys and increase data confidentiality, which contain time information and the key for the first time period. The packet is encrypted with the shared key of the source and destination nodes to prevent intermediate nodes from accessing it. By applying the hash function to the initial value of the hash chain, Max_Hash is calculated. The source node calculates this and places it inside the routing packet. The Hop_Count value is authenticated by intermediate nodes using this value. The source node sends the routing packet to the nodes within its radio radius (Eq 14):

$$S\to Brdcast:[RDP,N_{s},IP_{D},TTL,Max\_hash,{\left[P\right]}_{K\_SD},Hop\_Count,h_{0}]$$ (14)

The value of the hash chain is first processed by each intermediate node that receives this packet. After that, they calculate the new value of the hash chain. Also, the MAC value is calculated using the shared keys between itself and the source and placed inside the routing packet. In Fig 3, node N_1_ receives the routing packet, so this node first authenticates the hash chain (Eq 15):

$$h^{TTL-Hop\_Count}(h_{0})=Max\_Hop\_Count$$ (15)

If the validation is correct, it calculates the following values and sends the routing packet to the neighboring nodes (Eqs 16, 17 and 18):

$$h_{1}=h(h_{0})$$ (16)

$$H_{1}=MAC_{K\_N_{1}-s}\{RDP,N_{s},IP_{D},TTL,h_{1}\}$$ (17)

$$N_{1}\to Brdcast:[RDP,N_{s},IP_{D},TTL,Max\_Hash,\;{\left[P\right]}_{K\_SD},Hop\_Count,h_{1},N_{1},H_{1}]$$ (18)

As a one-hop neighbor of node N_1_, node N_3_ receives the route request packet and checks the hop count using the hashing chain. When this check is successful, the new hash chain and MAC values are calculated and sent to neighboring nodes (Eqs 19, 20, 21 and 22):

$$h^{\text{TTL-Hop\_Count}}(h_{0})=Max\_Hop\_Count$$ (19)

$$h_{2}=h(h_{1})$$ (20)

$$H_{2}=MAC_{K\_N_{3}-S}\{RDP,N_{s},IP_{D},TTL,h_{2},H_{1}\}$$ (21)

$$N_{3}\to Brdcast:[RDP,N_{s},IP_{D},TTL,Max\_hash,{\left[P\right]}_{K\_SD},Hop\_count,h_{2},(N_{1},N_{3}),(H_{1},H_{2})]$$ (22)

Until the route request packet reaches its destination, this process continues. In the end, the following packet reaches the destination node (Eq 23):

$$\left[RDP,N_{s},IP_{D},TTL,Max\_hash,{\left[P\right]}_{K\_SD},Hop\_count,h_{3},(N_{1},N_{3},N_{8}),(H_{1},H_{2},H_{3})\right]$$ (23)

### 5.2. Route response phase in SGHRP protocol

In this phase, the destination node authenticates the hop count using the hash chain, and if it is correct, it extracts the [P]_K_SD_ packet and decodes it to obtain the information about increasing confidentiality. Next, it constructs the route response packet as Eq 24 and sends it to the source node along the same route as the route request packet:

$$D\to N_{8}:[RDP,N_{D},IP_{D},(N_{1},N_{3},N_{8}),(H_{1},H_{2},H_{3}),H_{D}]$$ (24)

*H*_*D*_ is equal to the MAC calculated by the destination node based on the shared key between itself and the source node (Eq 25):

$$H_{\text{D}}={\text{MAC}}_{{\text{K}}_{\text{SD}}}\{RRP,N_{D},IP_{D},(N_{1},N_{3},N_{8}),(H_{1},H_{2},H_{3})\}$$ (25)

The intermediate nodes, after receiving the route response packet, put the shared keys with the source node with which they calculated the MACs into the route response packet. route response packets reach the source node based on the following relationships (Eqs 26, 27 and 28).


$$N_{8}\to N_{3}:[RRP,N_{D},IP_{D},(N_{1},N_{3},N_{8}),(H_{1},H_{2},H_{3}),(K_{N1-S}),H_{D}]$$ (26)

$$N_{3}\to N_{1}:[RRP,N_{D},IP_{D},(N_{1},N_{3},N_{8}),(H_{1},H_{2},H_{3}),(K_{N8-S,}K_{N3-S}),H_{D}]$$ (27)

$$N_{1}\to S:[RRP,N_{D},IP_{D},(N_{1},N_{3},N_{8}),(H_{1},H_{2},H_{3}),(K_{N8-S,}K_{N3-S,}K_{N1-S}),H_{D}]$$ (28)

Upon receiving the route response packet, the source node processes the MAC value created by the destination node (*H*_*D*_) using the shared key between it and the destination node, then extracts the shared keys with intermediate nodes from the route response packet and H_i_ (authenticates MAC values generated by intermediate nodes). The source and destination nodes are connected if the route response packet information is correct.

### 5.3. Route maintenance phase in SGHRP protocol

**Fig 4:**
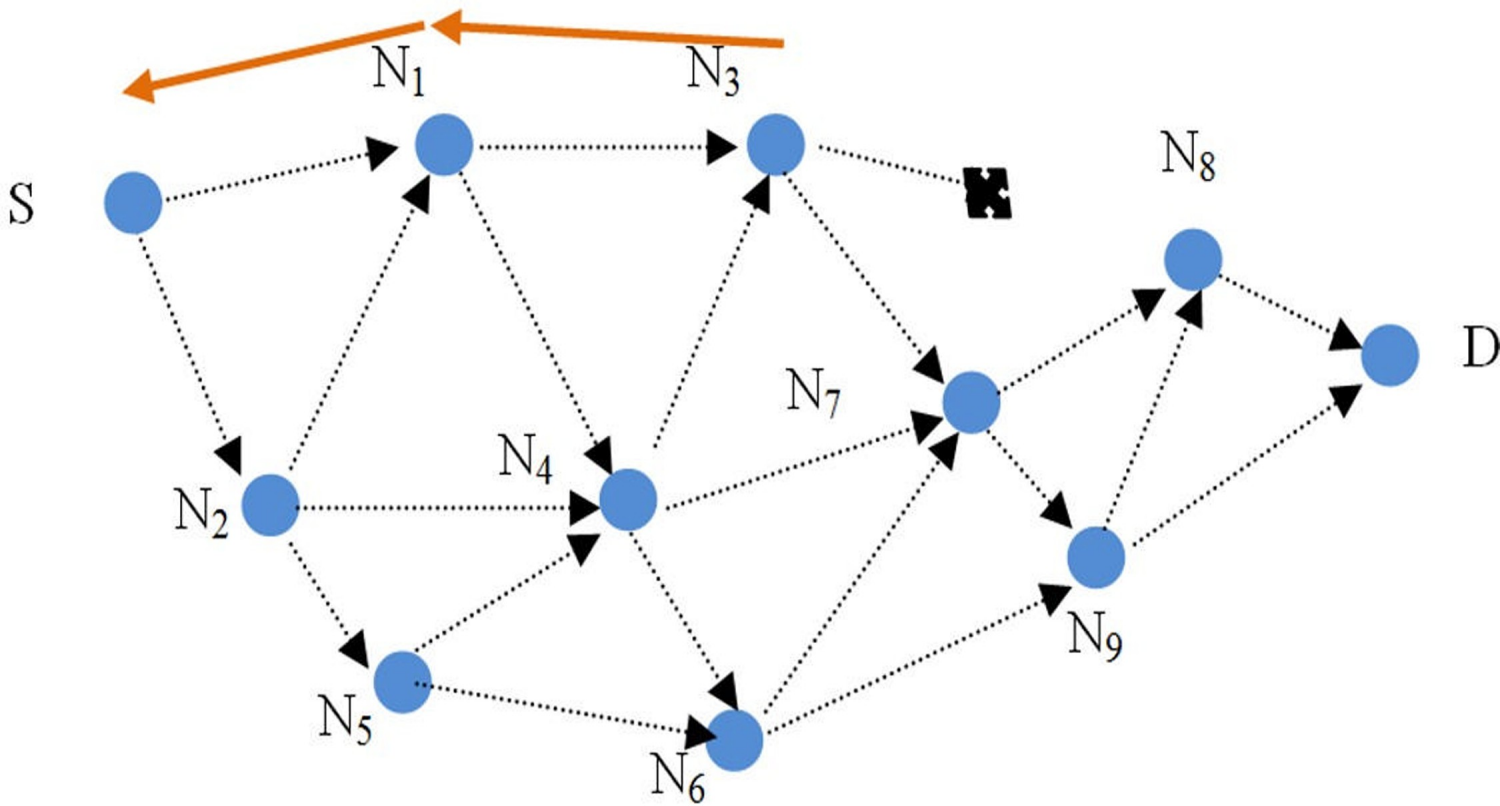
The intermediate nodes break the route, sending the route error packet.

The created route will be broken if the nodes move and leave each other’s radio range. The intermediate nodes are responsible for notifying the source node of this matter so that it can repair or reroute the route. Route error packets must also be authenticated to the source node in order to prevent tampering. In Fig 4, node N8 is assumed to have moved out of the radio range of node N3. N3 creates the route error packet as follows and sends it to the source node through the route (Eq 29):

$$N_{3}\to {\text{N}}_{1}:[\text{ERR},{\text{IP}}_{\text{s}},{\text{MAC}}_{{\text{K\_N}}_{\text{3}}\text{-S}}({\text{ERR,IP}}_{\text{S}}{\text{,IP}}_{N3}),K_{\text{N3-S}}]$$ (29)

In this packet, the MAC value is for validating the sender of the error packet, which is calculated by the shared key between the source and the sender of the error packet, and to prevent sending fake error packets, the sender node is authenticated for the source and intermediate nodes and it is stored inside the package.

### 5.4. SGHRP protocol confidentiality enhancement phase

**Fig 5:**
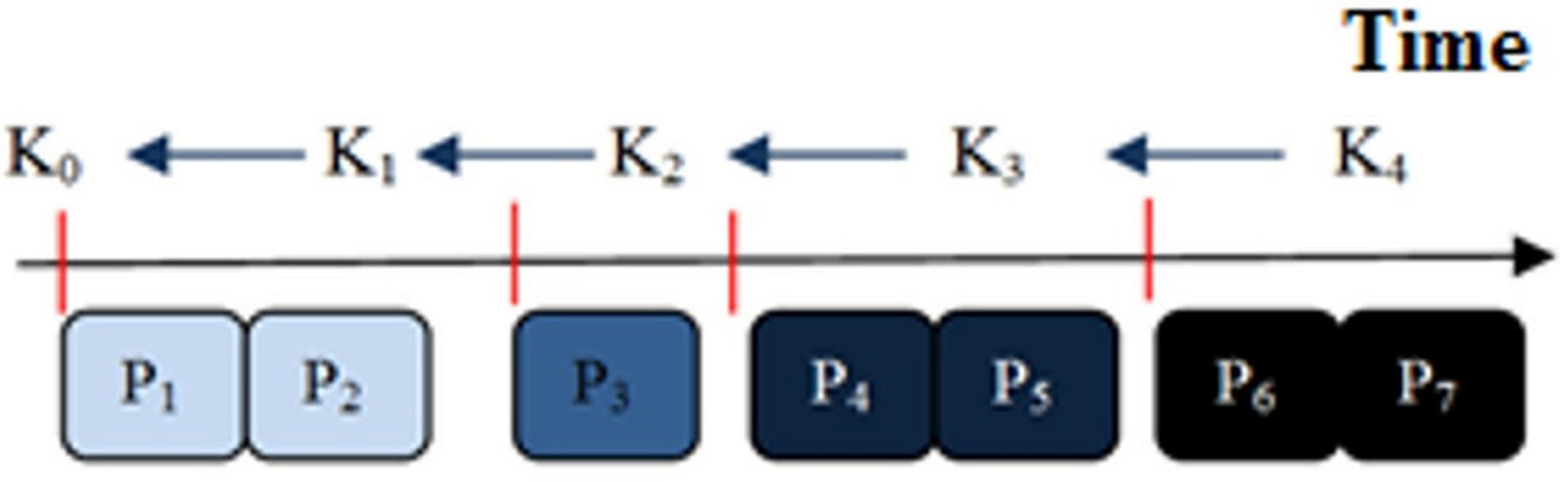
Encrypting and decrypting packets with one-way function keys [26].

After a route is created, most routing protocols do not pay attention to data confidentiality. By using a chain of source keys validated by a one-way function, the introduced protocol has provided increased confidentiality. A one-way function is used to validate the keys in this section, and then these keys are used to increase data confidentiality. To ensure that a receiver cannot forge the sender’s packets if it is attacked, an asymmetric mechanism should be used to verify the transmission of packets. However, this mechanism requires high overhead and requires high operational processing. For this purpose, the function One-way has been used, which by validating the symmetric keys somehow accesses the features of the asymmetric mechanism while improving data confidentiality as well. According to Fig 5, the K_1_ key is used to encrypt the P_1_ and P_2_ packets that arrived in the first time interval. First, the key K_1_ is validated by the one-way function (*K*_0_ = *F*(*K*_1_)), and if it is correct, the packets are decrypted. In order to decode packets related to the next interval, first the key related to that interval is validated by the one-way function in *K*_*i*_ = *F*^*j-i*^(*K*_*j*_), and then the packets are decoded by j>i [26]. The network member nodes hold the primary key K_0_ here. K_i_ (i>0) keys cannot be obtained using one-way hashing. K_i_ is the only way to validate them. As a result, instead of revealing the key with each package, we have done it independently.

When routing is being performed, the packet P, which is encrypted with the shared key between the source and destination, is sent to the receiving node along with the route request packet (Eq 30):

$$P=[T_{NOW},k_{i},T_{i},T_{int}]$$ (30)

Since all nodes have the key K_0_ of the source, the destination validates the key k_i_ by applying the one-way function (Eq 31):

$$K_{0}=F^{i}(K_{i})$$ (31)

In this case, the authentication key is used to decrypt packets received in time interval i. In this time interval, packets are sent using Eq 32.


$$S\to D:[{\left[M\right]}_{k_{i}}]$$ (32)

Due to the fact that the nodes are almost simultaneous, once the time period i has passed, the key for this period becomes invalid. At this time, the source node sends the key of the next period to the destination (Eq 33).


$$S\to D:{\left[T_{NOW},k_{i+1}T_{i+1},T_{int}\right]}_{K\_SD}$$ (33)

**Fig 6:**
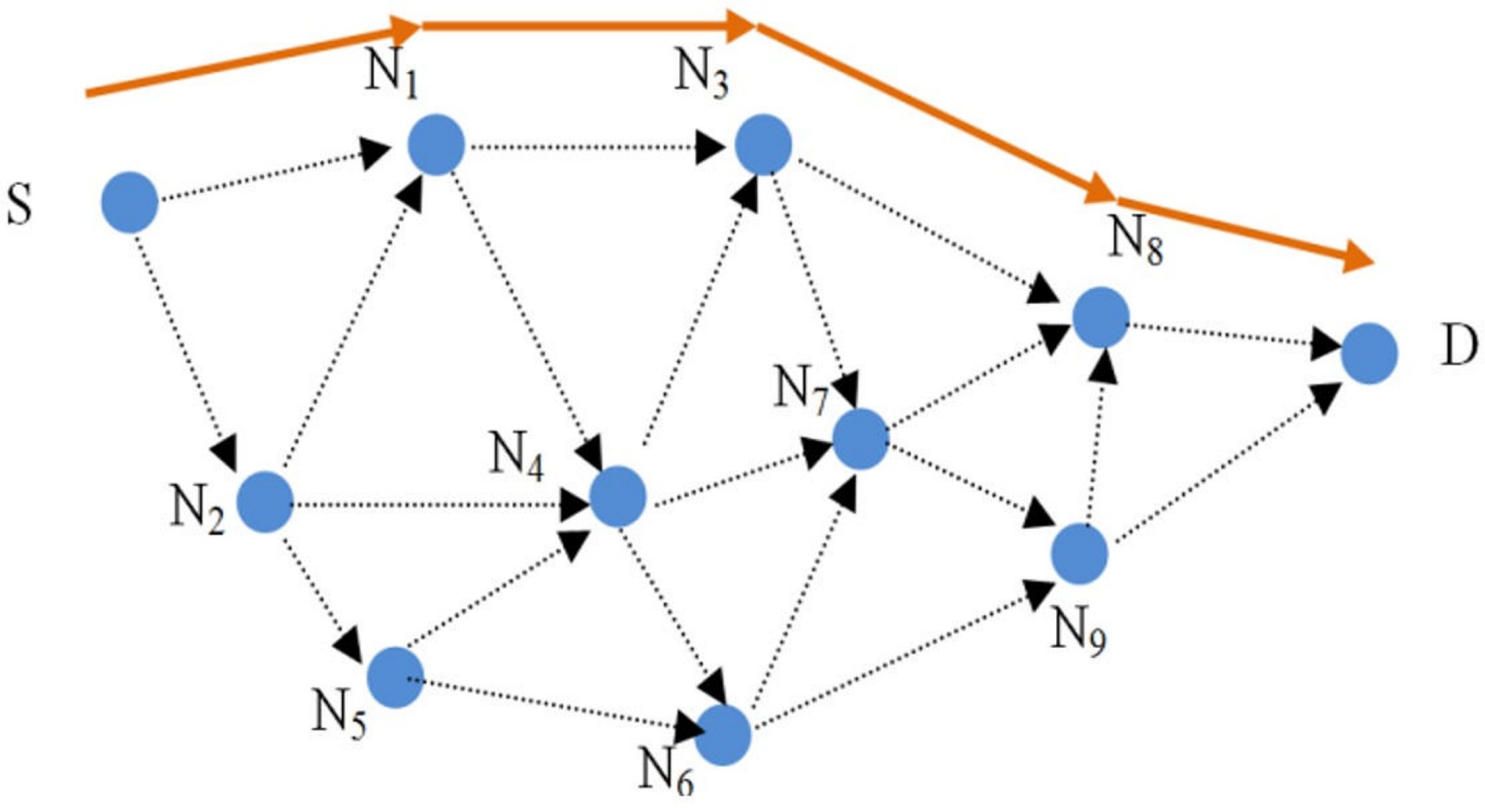
Sending time information and updating session keys.

To decrypt packets of period i+1, the destination node validates the key of period i+1 using the one-way function. The process will continue for the next period of time. The key can be sent in different time periods as shown in Fig 6.


$${\left[T_{NOW},K_{i+1},T_{i+1},T_{int}\right]}_{\text{K\_SD}}$$

## 6. Result and discussion

An example of a common attack in vehicular ad hoc networks routing called black hole is simulated here in order to test the protocol’s resistance against routing attacks. NS2 simulator software was used to simulate the proposed protocol and compare its performance parameters with those of other protocols. An average of 20 simulations is used to calculate all simulation results. The simulation parameters are shown in Table 2.

**Table 2:** Simulation parameters.

Parameters	Value
Network simulator	NS2
Simulation Time	1000s
Highway length	8 Km
Vehicles speed (min)	20 m/s
Vehicles speed (max)	33 m/s
Number of Vehicles	25, 50, 100, 200
Phy/Mac protocol	IEEE 802.11p
Data Packet Size	512 Bytes
Traffic	CBR
Transmission Range	250 m
Transmission Power	1 Mw

### 6.1. Performance parameters

Four parameters were evaluated to evaluate the efficiency of the proposed protocol and compare it with other algorithms: packet delivery rate, overhead, average end-to-end delay, and number of lost packets. Using these parameters, all four proposed protocols, SAODV, non-aggressive GHRP, and Blackhole GHRP (BGHRP), are compared.

#### 6.1.1. Packet delivery rate

According to Eq 34, the ratio of the total packets received by the destination node to the total packets sent by the source is:

$$\text{PDR}\;\text{=}\;\frac{\text{Number}\;\text{of}\;\text{packet}\;\text{received}\;\text{by}\;\text{destination}}{\text{Number}\;\text{of}\;\text{packet}\;\text{sent}\;\text{by}\;\text{source}}$$ (34)

**Fig 7:**
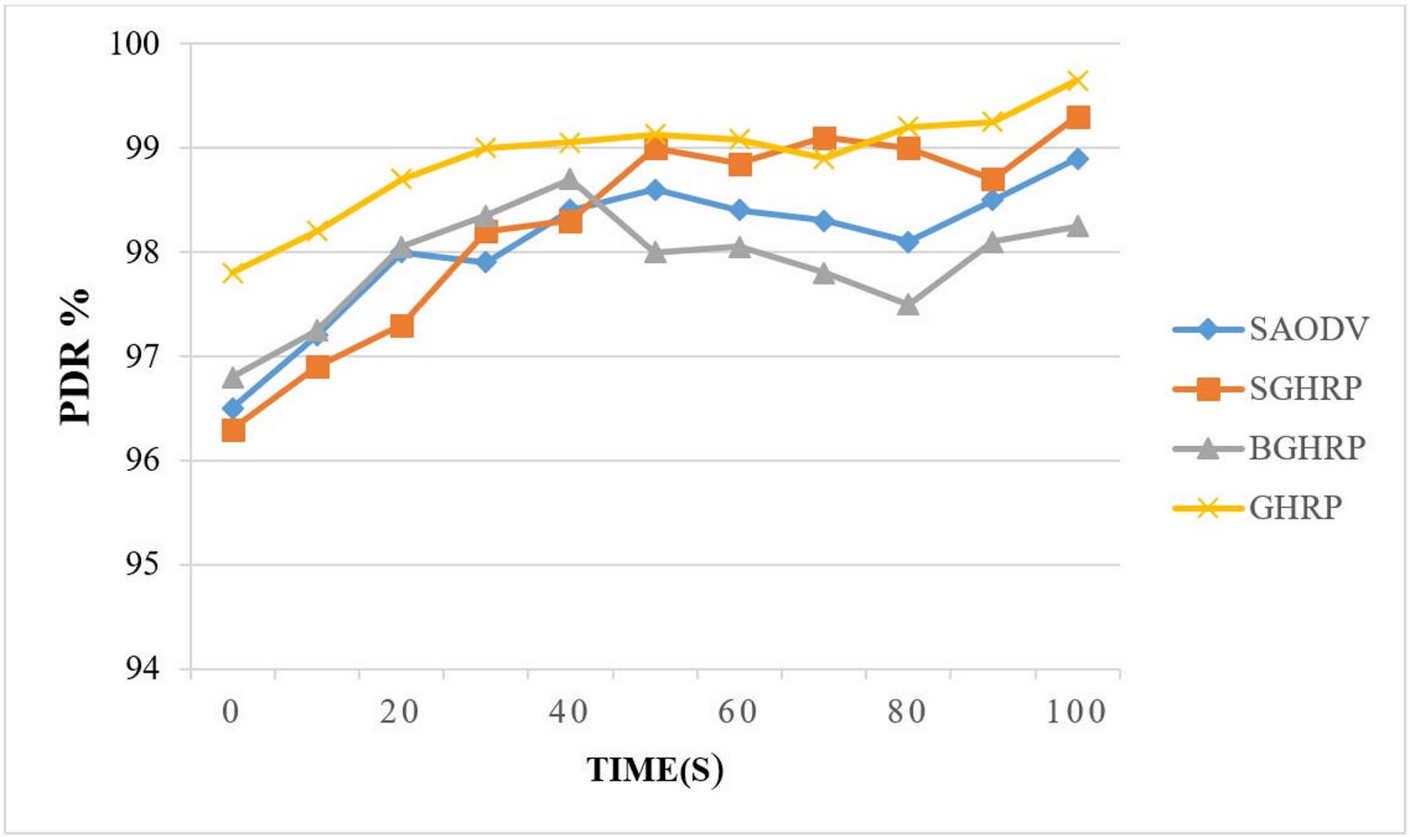
Package delivery rate.

Packet delivery rate informs the user about the protocol’s success rate when it comes to delivering data packets and routing at the application layer. When PDR is higher, the protocol has been more efficient in delivering packets. Fig 7 shows the effect of the attacking node and the mobility of the nodes on the packet delivery rate parameter.

In Fig 7, the PDR value for the proposed protocol is compared with the GHRP, BGHRP, and SAODV protocols over time. The graph shows that the PDR value for the proposed protocol in smaller times is close to the PDR value for SAODV protocols. As we get closer to the end of the simulation, this parameter appears more frequently in the proposed protocol and its value is higher than the SAODV protocol for times longer than 40 seconds. The key freshness does not seem to affect packet delivery rates much in less recent times, since the packets are encrypted with the primary key in the proposed protocol, and it is like using only one session key. Nevertheless, after the first period has passed, the session key between the source and destination nodes is changed, and packets sent and received are encrypted using the new key, reducing the likelihood of the key being leaked and increasing the number of packets received by the destination node.

#### 6.1.2. Normalized routing load

A normalized routing load (NRL) is the ratio of routing packets sent by a source to routing packets received by a destination and can be calculated using Eq 35.


$$\text{NRL}\;\text{=}\;\frac{\text{The}\;\text{number}\;\text{of}\;\text{routing}\;\text{packets}\;\text{sent}\;\text{by}\;\text{the}\;\text{source}}{\text{The}\;\text{number}\;\text{of}\;\text{data}\;\text{packets}\;\text{received}\;\text{by}\;\text{the}\;\text{destination}\;}$$ (35)

A higher value of NRL results in a lower efficiency and effectiveness of the protocol.

**Fig 8:**
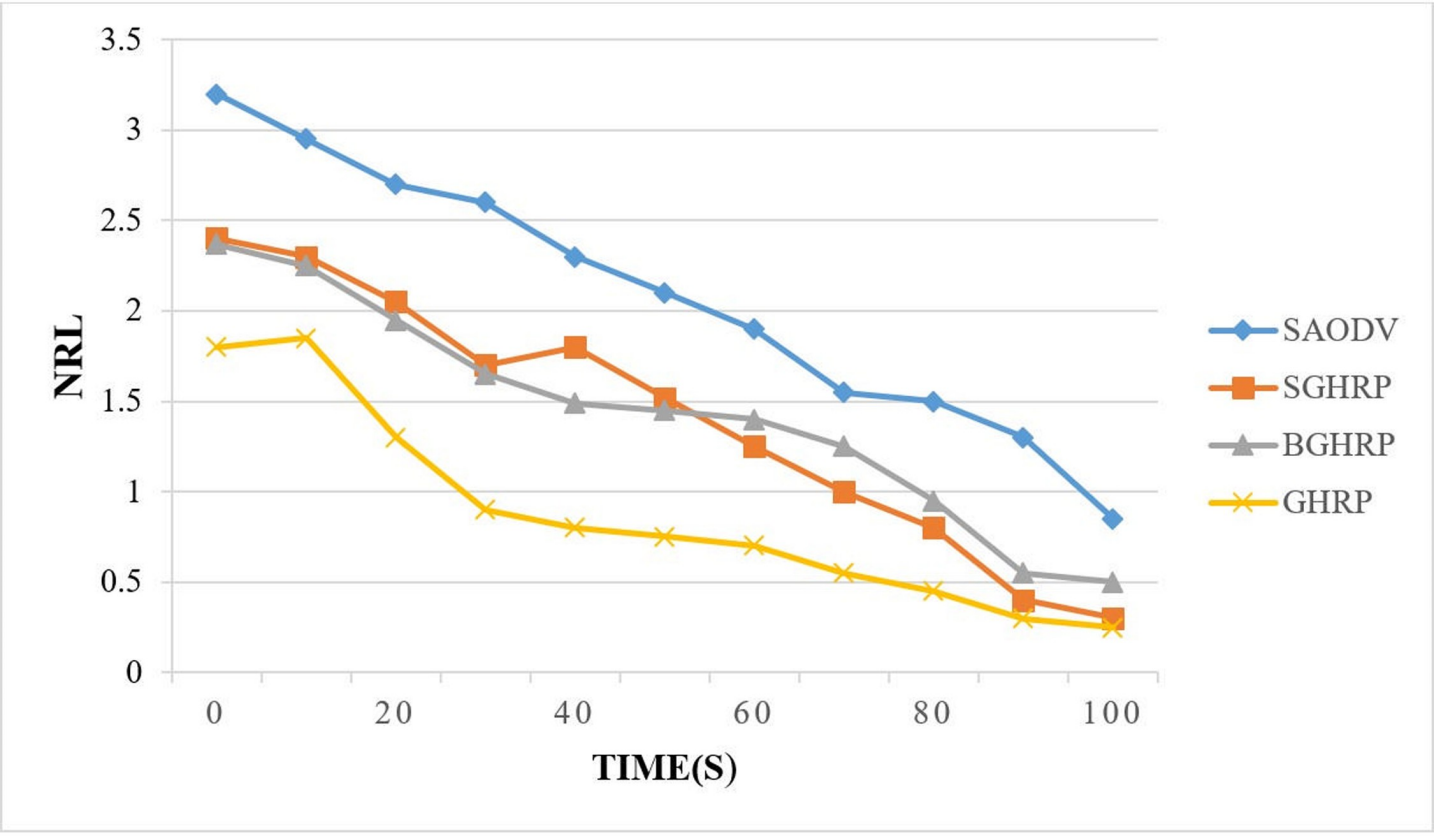
Routing overhead.

The routing overhead in the SGHRP protocol is higher than in the GHRP protocol because parts of the packets have been added to increase confidentiality. compares the normalized routing overhead between the proposed protocol and other protocols over time. According to the graph (Fig 8), the proposed protocol has a lower routing load than BGHRP and SAODV.

#### 6.1.3. Average end to end delay

The average end-to-end delay is the average delay between two end nodes during packet transmission. Eq 36 is used to calculate this parameter:

$$\text{AED}\;\text{=}\;\frac{\sum_{i=0}^{n}\;(The\;time\;of\;delivery\;of\;the\;ith\;package-The\;time\;of\;sending\;of\;the\;ith\;package)}{\text{The}\;\text{total}\;\text{number}\;\text{of}\;\text{packets}\;\text{received}\;\text{by}\;\text{the}\;\text{destination}\;}$$ (36)

**Fig 9:**
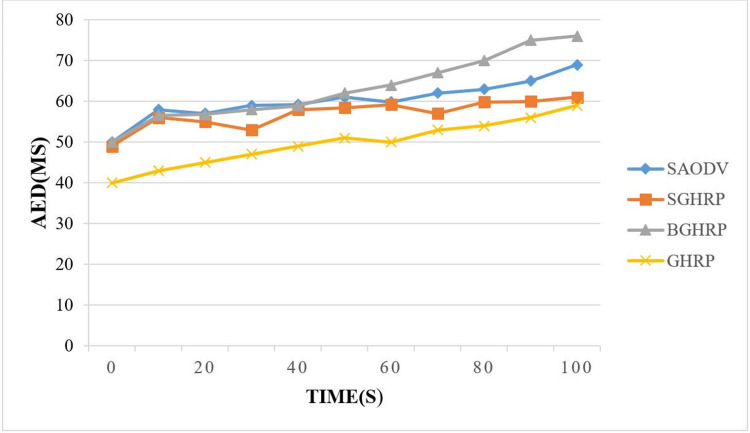
End to end delay.

Fig 9 shows the effect of the attacking node and the mobility of the nodes on the end-to-end delay parameter.

As soon as an attacker (black hole node) enters the route, routing is disrupted and packets are delayed in reaching their destinations. Because the black hole node drops routing packets, or the attacker eavesdrops on the packets and sends them to the destination, causing the packets to be delayed or not reach the destination, increasing the AED parameter. However, when the proposed protocols are used, according to the authentication, the routes that are created cannot be exploited. As a result, routing packets are not interrupted. Due to the presence of an attacker on short routes, the protocol may also have to use longer routes, which is why the packet arrival delay is higher than in GHRP.

#### 6.1.4. Number of dropped packets

During the simulation period, this parameter indicates the total number of packets that were lost and did not reach the destination. The number of dropped packets (NDP) can be calculated by using Eq 37:

NDP=Thenumberofpacketssent−Thenumberofpacketsdelivered (37)

**Fig 10:**
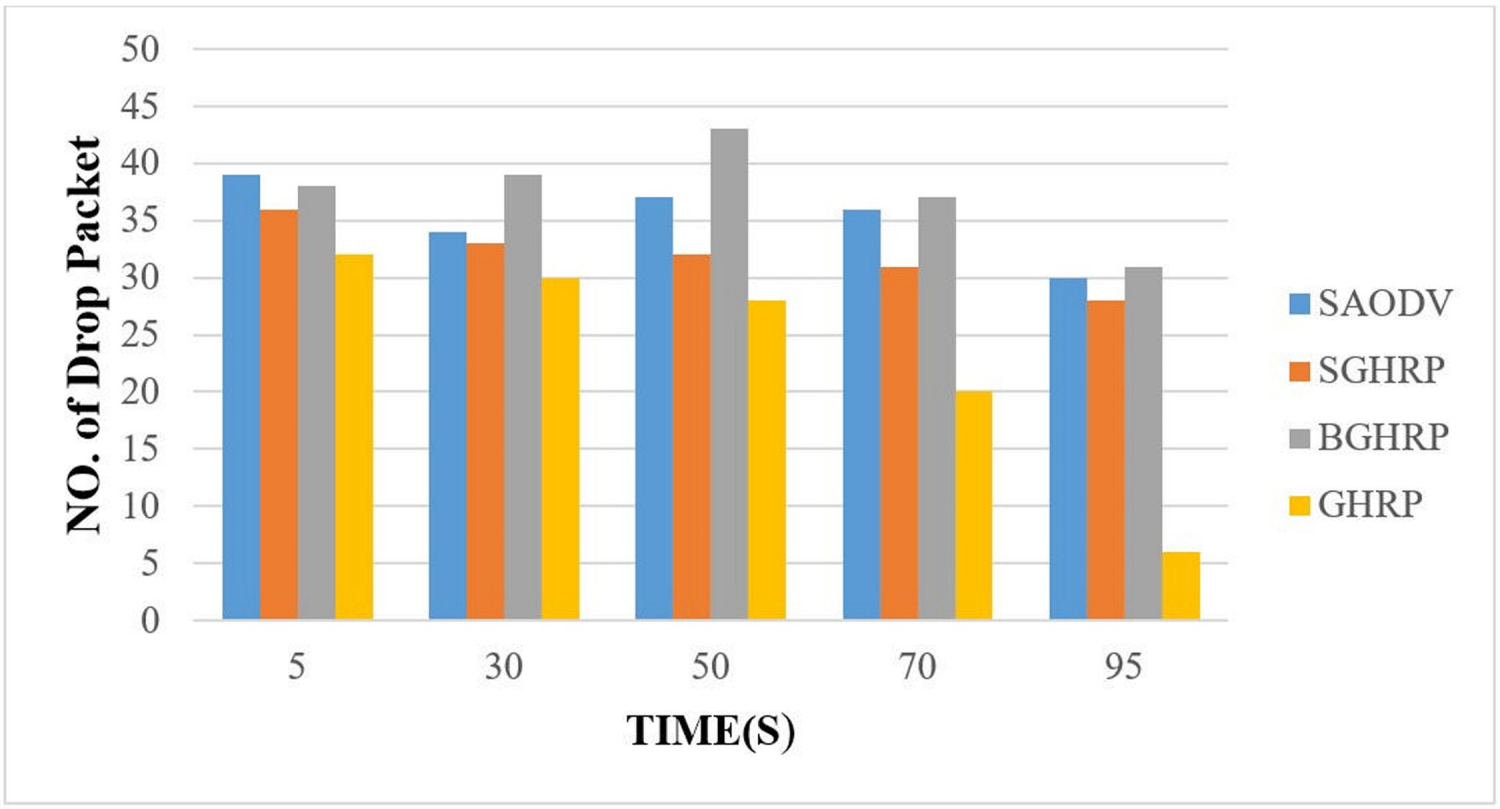
Packet loss percentage.

The presence of malicious nodes in the network increases the value of NDP and lowers the performance of the network. Fig 10 illustrates the effect of the attacking node on the number of lost packets as time increases.

Fig 10 shows the number of packet losses for different protocols at different times. The GHRP protocol has the best situation among the protocols. Although the proposed protocol has more losses than the GHRP protocol, they are relatively better than the BGHRP and SAODV protocols. The graph shows that packet loss has decreased over time, which is due to network stabilization.

## 7. Conclusion

The SGHRP protocol was proposed in this paper to authenticate the nodes, achieve high confidentiality, and reduce routing overhead and end-to-end delay. The proposed protocol has been implemented using NS2 software. When an attacker (black hole node) is present in the route, the routing is interrupted, causing packets to be delayed in reaching their destinations. As long as the proposed protocols are used according to authentication, the routes that are created will not be attacked, so the routing will not be interrupted and the end-to-end delay will be reduced. To achieve low routing overhead, hashing functions were used in the first stage to achieve security goals, and one-way hashing functions were used in the second stage to validate time interval keys. As we approach the end of the simulation, this parameter becomes apparent and increases in the proposed protocol. At low times, the PDR value for the proposed protocol is similar to the PDR values in SAODV and BGHRP. Additionally, package losses were compared. Although the proposed protocol has a higher loss than the GHRP protocol, it has a relatively better condition than SAODV and BGHRP. The black hole attack was tested as an example of routing attacks in the protocol evaluation section. Multiple nodes interacting in routing lead to other attacks, such as wormholes, in VANET networks. Protocols can be examined in terms of their resistance to such attacks.
